# Resistance to immune checkpoint inhibitors in advanced gastro-oesophageal cancers

**DOI:** 10.1038/s41416-021-01425-7

**Published:** 2021-07-06

**Authors:** Mark A. Baxter, Fearghas Middleton, Hannah P. Cagney, Russell D. Petty

**Affiliations:** 1 Division of Molecular and Clinical Medicine, Ninewells Hospital and Medical School, University of Dundee, Dundee, UK; 2 Tayside Cancer Centre, Ninewells Hospital and Medical School, NHS Tayside, Dundee, UK; 3 School of Medicine, Ninewells Hospital and Medical School, University of Dundee, Dundee, UK

**Keywords:** Gastric cancer, Oesophageal cancer

## Abstract

Immune checkpoint inhibitors (ICIs) have altered the treatment paradigm across a range of tumour types, including gastro-oesophageal cancers. For patients with any cancer type who respond, ICIs can confer long-term disease control and significantly improve survival and quality of life, but for patients with gastro-oesophageal cancer, ICIs can be transformative, as durable responses in advanced disease have hitherto been rare, especially in those patients who are resistant to first-line cytotoxic therapies. Results from trials in patients with advanced-stage gastro-oesophageal cancer have raised hopes that ICIs will be successful as adjuvant and neoadjuvant treatments in early-stage disease, when the majority of patients relapse after potential curative treatments, and several trials are ongoing. Unfortunately, however, ICI-responding patients appear to constitute a minority subgroup within gastro-oesophageal cancer, and resistance to ICI therapy (whether primary or acquired) is common. Understanding the biological mechanisms of ICI resistance is a current major research challenge and involves investigation of both tumour and patient-specific factors. In this review, we discuss the mechanisms underlying ICI resistance and their potential specific applications of this knowledge towards precision medicine strategies in the management of gastro-oesophageal cancers in clinical practice.

## Background

In the past decade, immune checkpoint inhibitors (ICIs) have reshaped the treatment landscape for many cancers. ICIs target cell-surface ‘immune checkpoints’—immune inhibitory pathways that normally function to mediate self-tolerance but that can be exploited by tumour cells to evade the host immune response.^1^ Indeed, the best recognised mechanism of evasion of the immune system by tumour cells is their upregulation of the immunosuppressive cell-surface ligands programmed cell death ligand-1 (PD-L1) and PD-L2. These ligands interact with the T-cell surface protein programmed cell death-1 (PD-1), resulting in suppression of T-cell activity via intracellular signalling. The expression of PD-L1 and PD-L2 has prognostic value across a range of tumour types, including gastro-oesophageal cancer. PD-L1 is reported to be expressed by 10–30% of gastro-oesophageal cancer cells,^2–4^ and the expression of PD-L1 and PD-L2 is associated with poorer survival in the neoadjuvant setting.^5^

The most striking example of the impact of ICIs is seen in metastatic melanoma, where the anti-PD-1 antibodies nivolumab and pembrolizumab and the anti-cytotoxic T lymphocyte antigen-4 (anti-CTLA-4) antibody ipilimumab have, within a decade, helped to extend the median survival of patients in clinical trials from less than a year^6^ to up to 5 years currently.^7,8^ This change in outcome has been driven by durability of response, which is the hallmark of these agents (unlike the benefit of other therapies, which tend to diminish with increased time). Despite this success, not all patients or cancer types benefit from ICIs and, in those that do, relapse can and does occur—even in patients who have shown a prolonged response. This phenomenon is known as resistance and can be classified as primary or acquired.^9^ Several common cancers, including gastro-oesophageal cancer, have an initial low frequency of response to ICIs (i.e. they show primary resistance); the rates of primary resistance can be estimated from response rates to ICI agents. The exact rates of acquired resistance, however, are poorly reported, and are complicated by the disputed occurrence of ‘hyperprogression’ and ‘pseudoprogression’ in some patients^10^ (Box 1).

Gastric cancer and oesophageal cancer are the fifth and eighth, respectively, most common cancers globally, and together cause an estimated 1.25 million deaths worldwide each year.^11^ Gastro-oesophageal cancer refers to cancer of the lower oesophagus, gastro-oesophageal junction and proximal stomach. The location of the primary lesion determines whether the cancer is considered and staged as gastric or oesophageal origin. In clinical settings, gastro-oesophageal cancer is currently classified according to histological subtype—the main subtypes are squamous cell carcinoma (SCC) and adenocarcinoma—and site of primary lesion.

Not only are SCC and adenocarcinoma associated with divergent histology, but also with different biology and aetiological factors; their pathogenesis and molecular biology must therefore be considered separately. Oesophageal SCC predominates in the upper and mid-oesophagus and is associated with smoking and alcohol, with a 5-year survival of ~15%.^12^ Gastro-oesophageal adenocarcinoma—in particular, adenocarcinoma of the lower oesophagus and gastro-oesophageal junction—is rapidly increasing in incidence in developed countries.^13^ It has a similarly poor 5-year survival to SCC (15–20%),^14,15^ and median survival in unselected patients in the advanced setting is less than a year.^16^

The mainstay of first-line treatment in patients with advanced gastro-oesophageal cancer is platinum-based chemotherapy, with agents such as docetaxel, paclitaxel and irinotecan used following progression. For cases of advanced gastro-oesophageal SCC or adenocarcinoma that are refractory to chemotherapy, durable responses have been seen using ICIs (18.0 months median duration of response in KEYNOTE-061^17^) but most patients (~85%) have primary resistance and do not benefit from ICI monotherapy. Those who do respond often develop acquired resistance. Accurately identifying subgroups of patients with gastro-oesophageal cancer who are likely to benefit significantly from ICIs is a therapeutic challenge. Currently, biomarkers for response to ICIs include the extent of microsatellite instability, PD-L1 expression and, potentially, presence of the Epstein–Barr virus (EBV). Alongside identifying biomarkers to predict response, research has also focused on uncovering mechanisms of resistance to ICIs, but identifying such mechanisms is challenging in gastro-oesophageal cancer owing to the complex disease biology.^18,19^ In this review, we discuss the clinical impact of ICIs on the treatment of gastro-oesophageal cancer thus far, examine mechanisms of resistance, and explore how resistance can potentially be circumvented in order to effectively treat patients.

Box 1 Definitions of types of tumour response seen with immune checkpoint inhibitors. **Type of response Definition**.

**Hyperprogression** Rapid increase in rate of tumour progression on commencing ICI therapy.

**Primary resistance** No response to initial therapy.

**Acquired resistance** Initially response to ICI observed but after a period of time progression seen.

**Adaptive resistance** Cancer is recognised by the immune system but protects itself by adapting to the immune attack; can manifest as primary or acquired resistance.

**Pseudoprogression** Initial growth in tumour burden followed by a response.

## Molecular characteristics of gastro-oesophageal cancer

The clinical classification of gastro-oesophageal cancer was outlined above; work carried out over the past decade has focused on characterising specific molecular subtypes. This work suggests that there could be specific molecular subgroups of both gastric and oesophageal adenocarcinomas that might be sensitive to ICIs, and that oesophageal SCC needs to be considered separately.^20^

### Gastric cancer

The Cancer Genome Atlas (TCGA) has profiled gastric cancer into four subtypes: EBV (9%); microsatellite instability (MSI; 22%); genomically stable (GS; 20%); and chromosomal instability (CIN; 50%).^21^ The subtypes appear to have prognostic value, with EBV having the best prognosis and GS having the worst.^22^ Within the TCGA cohort, the EBV subtype was enriched for amplification of the genes that encode PD-L1 and PD-L2 and, with the link between MSI, CD8^+^ T-cell infiltration and ICI response now being well recognised,^23,24^ it is therefore likely that both the EBV and MSI subgroups might be susceptible to ICIs. By contrast, GS tumours appear to be immune evasive, and the CIN subtype is associated with amplification of receptor tyrosine kinases (RTKs) and high levels of somatic copy number change. Several of these RTKs have been linked to the local immune landscape and are candidates for molecularly targeted therapies. Further analysis of the immune infiltrate (a high degree of macrophage infiltration with T-cell exclusion) and interferon-γ (IFN-γ) levels (low levels) showed that CIN tumours were most likely to be immunologically evasive^22^ and respond poorly to ICIs.^20^

### Oesophageal cancer

#### Oesophageal SCC

On the basis of molecular features, oesophageal SCC more closely resembles SCC of other organs than it does oesophageal adenocarcinoma. This observation might have therapeutic implications as oesophageal SCC and adenocarcinoma have historically been treated with similar systemic regimes—this approach may need to change. Oesophageal SCC has three molecular subclasses: ESCC1, which is associated with alterations in the nuclear factor erythroid 2-related factor 2 (NRF2) pathway and is present in an East Asian population; ESCC2, which is associated with a NOTCH1 mutation and is present in an Eastern European and South American population; and ESCC3, which is associated with activation of the phosphoinositide 3-kinase (PI3K) pathway and is predominant in a North American population.^12^

#### Oesophageal adenocarcinoma

An integrated genomic analysis of 164 oesophageal cancers found that oesophageal adenocarcinoma strongly resembled the CIN variant of gastric cancer, with some variation in molecular features such as hypermethylation.^25^ Further work by the UK Oesophageal Cancer Clinical and Molecular Stratification (OCCAMS) group on 129 oesophageal and gastro-oesophageal junction adenocarcinomas defined three mutational signatures: a BRCA-like signature (15%); a mutagenic signature with significant neoantigen load and tumour mutational burden (TMB) (53%); and a C > A/T signature associated with ageing (32%).^18^ The OCCAMS consortium subsequently used combined multi-omic characterisation of 551 oesophageal adenocarcinoma samples to identify 77 driver genes with a mean of 4.4 driver events per tumour, which are commonly derived from mutations rather than copy number events. These events are often associated with numerous exclusive or co-occurring dysregulated signalling pathways, highlighting the challenge heterogeneity brings to identifying a specific therapeutic target.^19^ Although felt to genomically resemble the immune evasive CIN variant of gastric cancer, oesophageal adenocarcinoma has a higher TMB compared with gastric cancer and other tumour types, with 9.9 mutations/Mb, ranked behind only those cancers in which ICIs have been approved (e.g. melanoma, lung and bladder cancer).^26^

## Current impact of ICIs in advanced gastro-oesophageal cancers

ICIs have shown promising activity in gastro-oesophageal cancer (Table 1), with response rates in pre-treated patients ranging from 11 to 24%, depending on the combined positivity score (CPS; the ratio of cells (both tumour and immune) expressing PD-L1 relative to the number of viable tumour cells).^17^ The response rates appear constant regardless of line of therapy,^17,27,28^ which suggests that a distinct population of patients who will benefit exists. When responses occur, they often prove durable. Currently, the best predictive biomarkers of response are the extent of MSI and PD-L1 expression.^28^ However, although response rates are in excess of 50% in MSI-high tumours, these tumours constitute only ~4% of gastro-oesophageal cancers.^28^ For this reason, much research has focussed on the expression of PD-L1. Interestingly, several antibodies for immunohistochemistry exist; their varying specificities and sensitivities^29^ are likely to account for the range of expression estimates and might also influence the ability to accurately quantify a patient’s tumour expression and therefore eligibility for therapy. PD-L1 expression has a good negative predictive value for response (negative expression is associated with a response rate (RR) of 2–6% for ICI monotherapy) but a poorer positive predictive value (a PD-L1 CPS ≥ 1 is associated with a RR of 15–16%, and a PD-L1 CPS ≥ 10 is associated with a RR of 24–25%). There is a need for better biomarkers to drive personalised treatments.

**Table 1:** Practice-changing Phase 3 trials in advanced gastro-oesophageal cancer involving immune checkpoint inhibitors.

Trial	Phase	Population	Setting	Agent	Response rate	PFS	OS
*>2nd line*							
ATTRACTION-2 Kang at al.^34^	3	Advanced GOJ or GC (*n* = 493) Asian population	≥2nd line	Nivolumab (*n* = 330) vs placebo (*n* = 163)	11% (8–16%) DCR 40% (34–46%)	1.61 m (1.5–2.3)	5.3 m vs 4.1 m (HR 0.63, *P* < 0.0001)
KEYNOTE-059 (cohort 1) Fuchs et al.^28^	2	GC/GOJ (*n* = 259)	≥2nd line	Pembrolizumab (all patients)	12%^8–17^ PD-L1 + ve 16% PD-L1 –ve 6% DCR 27^22–33^	2.0 m (2.0–2.1)	5.5 m (4.2–6.5)
CHECKMATE-032 Janjigian et el.^37^	1/2	Advanced OC, GOJ or GC (*n* = 160)	≥2nd line	Nivolumab 3 mg/kg (*n* = 59) nivolumab 1 mg/kg + Ipilimumab 3 mg/kg (*n* = 49) nivolumab 3 mg/kg + Ipilimumab 1 mg/kg (*n* = 52)	12% vs 24% vs 8%	12 month: 8% vs 17% vs 10%	12 month: 39% vs 35% vs 24%
JAVELIN-300 Bang et al.^120^	3	GOJ (*n* = 111) or GC (*n* = 260)	3rd line	Avelumab vs TPC	2.2% vs 4.3% DCR 22.2 vs 44.1%	1.4 m vs 2.7 m	4.6 m vs 5.0 m (HR 1.1, NS)
*2nd line*							
KEYNOTE-061 Shitara et al.^17^	3	GOJ (*n* = 185) or GC (*n* = 407)	2nd line	Pembrolizumab (*n* = 296) vs paclitaxel (*n* = 296)	CPS ≥ 1: 16% vs 14% CPS ≥ 10: 24.5% v 9.1% CPS < 1: 2% vs 10.4%	1.5 m vs 4.1 m (HR 1.27) CPS < 1: HR 2.05	9.1 m vs 8.3 m (HR 0.82, *P* = 0.0421) CPS ≥ 10: 10.4 m v 8.0 m (HR 0.64) CPS < 1: 4.8 m vs 8.2 m (HR 1.20)
KEYNOTE-181 Kojima et al.^36^	3	Advanced OAC (*n* = 227) or OSCC (*n* = 401)	2nd line	Pembrolizumab vs TPC	–	–	ITT: 7.1 m vs 7.1 m SCC: 8.2 m vs 7.1 m CPS > 10: 9.3 m vs 6.7 m
*1st line*							
KEYNOTE-062 Tabernero et al.^27^	3	GC/GOJ (PD-L1 CPS ≥ 1%)	1st line	Pembrolizumab (P) (*n* = 256) pembrolizumab + chemotherapy (P + CTx) (*n* = 257) chemotherapy (CTx) (*n* = 250)	P v CTx: 14.8% vs 37.2% CPS ≥ 10: 25.0% vs 37.8%	P v CTx CPS ≥ 1: 2.0 m v 6.4 m (HR 1.66), CPS ≥ 10: 2.9 m v 6.1 m (HR 1.10)	P v CTx ITT: 12.5 m v 11.1 m (HR 0.85) CPS ≥ 1: 10.6 m v 11.1 m (HR 0.91) CPS ≥ 10: 17.4 m vs 10.8 m (HR 0.69)
ATTRACTION-4 Boku et al.^39^	2	GC/GOJ (*n* = 724)	1st line	Nivolumab (*n* = 362)+CTx (SOX/CapOX) vs CTx alone (*n* = 362)	58% vs 48%	10.5 vs 8.4 (HR 0.68)	17.5 m vs 17.2 (HR 0.90)
KEYNOTE-590 Kato et al.^41^	3	OAC or OSCC (*n* = 749)	1st line	Chemotherapy + /pembrolizumab	OSCC: 45% vs 29.3%	–	OSCC (all): 12.6 m vs 9.8 m (HR 0.72) OSCC (CPS ≥ 10): 13.9 m vs 8.8 m (HR 0.57) ITT 12.4 m vs 9.8 m
CHECKMATE 649 Moehler et al.^40^	3	GC/GOJ (*n* = 1266, including Ipilimumab/nivolumab arm)	1st line	Nivolumab+chemotherapy (*n* = 473) chemotherapy alone (*n* = 482)	CPS > 5: 60% vs 45%	CPS ≥ 5: 7.7 m vs 6.0 m (HR 0.68)	CPS ≥ 5: 14.4 m vs 11.1 m (HR 0.71, *P* < 0.0001)
*ICI maintenance*							
JAVELIN 100 Moehler et al.^4^	3	GC/GOJ (*n* = 805, of whom 499 entered 2nd phase)	1st line maintenance	Avelumab (*n* = 249) vs ongoing chemotherapy (*n* = 250)	13.3% vs 14.4% 1-year duration of response 62.3% vs 28.4%	HR 1.04 (0.85–1.28)	10.4 m vs 10.9 m (HR 0.91, *P* = 0.1779) 24–month OS 22.1% vs 15.5%

*DCR* disease control rate, *PFS* progression-free survival, *OS* overall survival, *HR* hazard ratio, *NS* non-significant, *m* month, *SOX* S-1/oxaliplatin, *CapOx* capecitabine/oxaliplatin, *GOJ* gastroesophageal junctional, *GC* gastric cancer, *OAC* oesophageal adenocarcinoma, *OSCC* oesophageal squamous cell carcinoma, *PD-L1* programmed death ligand 1, *TPC* treatment of physicians choice, *CPS* combined positivity score.

### ICIs in pre-treated patients

CTLA-4 inhibitors were the first ICIs to be trialled in advanced gastro-oesophageal cancer, but ipilimumab and tremelimumab both showed limited success in the Phase 2 setting.^30,31^ A Phase 1b/2 study with tremelimumab and the PD-L1 inhibitor durvalumab, either alone or in combination, again demonstrated low response rates.^32^ The first randomised Phase 3 trial to show promise using a single-agent ICI—the PD-1 inhibitor nivolumab—was ATTRACTION-2.^33^ In pre-treated patients with advanced gastric or gastro-oesophageal cancer, nivolumab conferred a survival advantage over placebo with a median overall survival of 5.3 months versus 4.1 months (hazard ratio [HR] 0.62, *P* < 0.0001), a response rate of 11% versus 0%, and 1-year survival of 27% versus 12%, suggesting a significant advantage for a subgroup of patients.

The KEYNOTE-061 trial compared pembrolizumab to paclitaxel in the second-line setting.^17^ Importantly, the PD-L1-negative subgroup was terminated because of a worse survival compared to paclitaxel, again highlighting the negative predictive value of low PD-L1 expression. In patients with a CPS ≥ 1, median progression-free survival (PFS) was inferior with pembrolizumab (1.5 months versus 4.1 months with paclitaxel, HR 1.27). Although median overall survival (OS) trended towards significance, the one-sided alpha of 0.0135 was not met (9.1 months versus 8.3 months, HR 0.82, *P* = 0.0421). Response rates ranged from 16% to 24%, depending on the CPS. Of note, the survival curves crossed, indicating the potential existence of two distinct populations—one that derived benefit from ICI therapy and the other that derived absolutely no benefit and accounted for the initial steep fall in the survival curve of the pembrolizumab arm. The existence of these distinct populations highlights the importance of choosing the correct treatment for individual patients.^34^

As highlighted above, oesophageal SCC differs biologically from oesophageal adenocarcinoma, yet appears to benefit from the use of nivolumab or pembrolizumab in the second-line setting. The ATTRACTION-3^35^ study demonstrated a survival advantage for nivolumab over chemotherapy (median OS 10.9 months versus 8.4 months; HR 0.77, *P* = 0.019), while the KEYNOTE-181 study demonstrated a survival advantage for pembrolizumab over chemotherapy in patients with SCC and a CPS ≥ 10 (median OS 10.3 months versus 6.7 months; HR 0.69).^36^ There was no survival advantage for oesophageal SCC CPS < 10 or adenocarcinoma regardless of CPS.

### ICIs in the first-line setting

Early-phase trials of combination immunotherapy in the later setting indicate marginal improvements in outcome, but at the risk of greater toxicity.^37^ Trials have subsequently moved to explore the role of ICIs in the first-line setting: as a single agent, or in combination with either chemotherapy or another ICI; and in maintenance therapy following induction chemotherapy.

#### ICIs alone or combined with another ICI or chemotherapy

The KEYNOTE-062 study^38^ explored the use of pembrolizumab or chemotherapy in combination or alone as first-line treatment of advanced gastric or gastro-oesophageal junction adenocarcinoma. Pembrolizumab alone was non-inferior to chemotherapy for survival in patients with CPS ≥ 1, albeit with a lower PFS and response rate. The survival curves, similar to those in KEYNOTE-061, again crossed. The greatest benefit of ICI therapy was seen in those with CPS ≥ 10 (median OS 17.4 months versus 10.8 months). However, the combination arm was not superior to chemotherapy alone in either OS or PFS, regardless of the CPS, and was associated with higher toxicity (73% of patients had grade 3–5 toxicities). The HR for the combination arm was 0.85 for both the CPS ≥ 1 (257 patients) and ≥10 arms (99 patients). The lack of significance might be partly explained by lowering the power of the study with co-primary endpoints and smaller patient numbers.

Similarly, the ATTRACTION-4 study investigated the use of nivolumab with chemotherapy in an Asian population including patients with gastric or oesophageal adenocarcinomas. There was a PFS benefit but no difference in OS between the groups (median OS 17.5 months versus 17.2 months; HR 0.9, *P* = 0.259). Many patients received subsequent lines of treatment on progression, including a high rate of crossover to ICIs, which might have influenced survival analysis.^39^ This might also provide further support for the evidence that ICIs can be effective regardless of line of therapy.

By contrast, the international CheckMate 649 study^40^ investigating the use of nivolumab with chemotherapy found a survival advantage conferred by combination therapy in the CPS ≥ 5 group (median OS 14.4 months versus 11.1 months, HR 0.71). OS was the primary endpoint and, importantly, 473 patients received combination therapy, which increased the study power.

The KEYNOTE 590 study^41^ investigated chemotherapy with or without pembrolizumab in the first-line setting for advanced gastro-oesophageal cancer (both oesophageal adenocarcinoma and SCC were included). In the SCC population, pembrolizumab conferred a survival advantage in the population as a whole (median OS 12.6 months versus 9.8 months, HR 0.72) but particularly in the CPS ≥ 10 group (median OS 13.9 months versus 8.8 months, HR 0.57).

#### ICIs in maintenance therapy following induction chemotherapy

Another therapeutic approach that has been investigated involves maintenance therapy with ICI after induction chemotherapy; for example, the JAVELIN-100 trial of avelumab, which targets PD-L1, versus continuation of chemotherapy.^4^ However, in this study, 38% of patients did not progress to the maintenance stage. In those who were randomised, no difference was observed in the primary endpoint of median OS in all patients or the response rate; however, the 2-year OS was improved, and the duration of response was longer for avelumab. A retrospective post-hoc analysis of 137 patients with CPS ≥ 1 demonstrated a median OS of 14.9 months versus 11.6 months (HR 0.72, 95% confidence interval (CI) 0.49–1.05), favouring avelumab. Avelumab, in contrast with nivolumab and pembrolizumab, targets PD-L1; there is some evidence that anti-PD-1 ICIs, which have shown benefit in gastro-oesophageal cancers, deliver different clinical outcomes to ICIs that target PD-L1,^42^ which might be an additional relevant factor in explaining the negative outcome of this trial. Ongoing studies, such as PLATFORM (NCT02678182), that are evaluating maintenance treatment with ICIs will help to address this issue, as well as clarify the role of maintenance treatment with ICIs more broadly in gastro-oesophageal cancers.

## Mechanisms of ICI resistance in gastro-oesophageal cancer

The trial results outlined above suggest that a combination regimen with chemotherapy and ICI should now be first line for patients with advanced oesophageal SCC and a CPS ≥ 10, or for those with advanced gastro-oesophageal or oesophageal adenocarcinoma and a CPS ≥ 5. However, they also highlight that not all patients benefit from ICI therapy, and the importance of understanding the underlying reasons for this resistance.

**Fig. 1:**
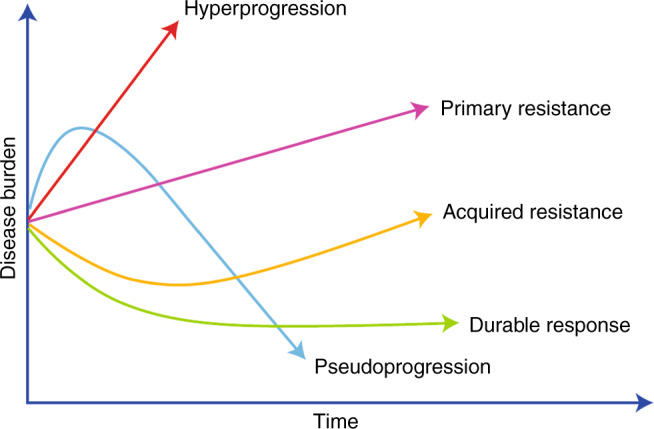
Patterns of disease response to immune checkpoint inhibitors. Disease burden is on the *x*-axis and time on the *y*-axis. All patients begin with a level of disease burden. The subsequent patient of disease response can fall into one of five broad categories; hyperprogression, primary resistance, pseudoprogression, acquired resistance or durable response.

**Fig. 2:**
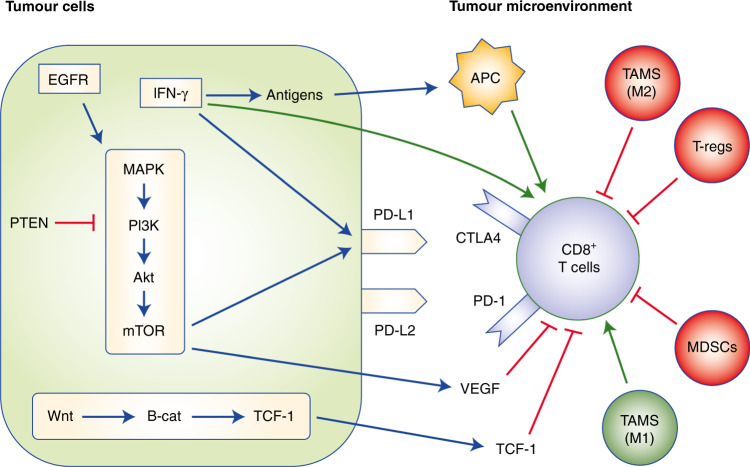
The impact of intracellular tumour cell pathways and the tumour microenvironment on cytotoxic T cells in gastro-oesophageal cancer. The figure depicts the various influences on cytotoxic T-cell function relating to the signalling pathways within the tumour itself as well as the tumour microenvironment (TME). EGFR epidermal growth factor receptor, IFNy interferon y, PTEN phosphatase and tensin homologue, MAPK mitogen-activated protein kinase, PI3K phosphoinositide 3-kinases, β-cat β-catenin, TCF-1 T-cell factor 1, APC antigen-presenting cell, VEGF vascular-endothelial growth factor, MDSCs myeloid-derived suppressor cells, TAM tumour-associated macrophages, PD-L1 programmed death ligand 1, PD-L2 programmed death ligand 2, CTLA-4 cytotoxic T lymphocyte antigen 4.

Resistance can be either primary or acquired (Box 1 and Fig. 1) and can involve both intrinsic signalling and the tumour microenvironment (TME) (Fig. 2). Involvement of the immune system in promoting resistance is termed ‘adaptive’. The interaction between host and tumour is constantly evolving, and resistance can manifest at any disease stage.

Some patients appear to experience a dramatic acceleration in disease progression following commencement of immune checkpoint blockade, termed hyperprogression, which is associated with very poor survival.^10^ This phenomenon must be distinguished from pseudoprogression, where a tumour response is seen following an initial apparent increase in disease burden (Box 1).

### Hyperprogression

Hyperprogression is a controversial topic, with opponents contending it is the natural history of the disease. Multiple definitions exist, but the underlying theme is the occurrence of a rapid escalation of tumour progression on commencement of ICI therapy.^43–45^ Retrospective analyses of large studies have shown rates of hyperprogression of 4–17% with ICIs (much higher than with chemotherapy); the effect is not tumour-specific and is associated with decreased PFS and OS.^45–47^ The underlying mechanism is unknown, but evidence points to a role for alterations in the gene that encodes the epidermal growth factor receptor (EGFR) and amplification of MDM2/4, in addition to the presence of M2-like macrophages, which promote tumorigenesis.^45,48^ Notably, EGFR alterations are present in 19% of oesophageal SCCs and 15% of oesophageal adenocarcinomas as an oncogenic driving mechanism.^12^

In the Asian ATTRACTION-2 study,^33^ which is the only study comparing ICI therapy with best supportive care only, the survival curves do not cross. If true hyperprogression was occurring, crossing of the survival curves might be expected as a subgroup of patients who received an ICI would experience an acceleration of their natural disease trajectory. This observation could suggest that what is perceived as hyperprogression in other studies comparing ICI therapy with an active control arm in gastro-oesophageal cancer might, in fact, be the result of a group of patients who derive absolutely no benefit from ICI therapy demonstrating the natural history of the disease. This natural history would appear worse than control arms of active chemotherapy and be similar to the response observed in other trials with best supportive care alone arms in this setting.^49^

### Primary resistance

Patients who do not respond to initial therapy have primary resistance. Although stable disease can be associated with patient benefit in some cases, considering that the majority of patients with advanced stage gastro-oesophageal cancer are often very symptomatic and have high tumour burdens, then ideally a reduction in tumour volume should be achieved. In this clinical context, if stable disease is considered as resistance, primary resistance accounts for ~85% of patients. Primary resistance is driven primarily by intrinsic tumoural cellular signalling and the TME. Host-related factors such as age, human leukocyte antigen (HLA) type, diet and the gut microbiome have also been postulated to contribute to resistance, but data are preliminary and no clear therapeutic targets have been identified.

### Intrinsic tumoural cell signalling

In gastro-oesophageal cancer, primary resistance to ICIs is driven by signalling involving a complex interplay of pathways including the extracellular signal-regulated kinase/mitogen-activated protein kinase (ERK/MAPK), WNT–β-catenin and IFN-γ signalling pathways, which together influence the cell-surface expression of immune checkpoint molecules. The end result is downregulation of T-cell function.

The MAPK pathway, which encompasses activation of RAS, RAF, MAPK and ERK kinase (MEK) and ERK/MAPK is upregulated in 52–60% of gastro-oesophageal adenocarcinomas.^50,51^ This pathway plays a critical role in cell fate decisions within CD8 + T cells including influencing proliferation and survival.^52^ In tumour cells, activation of the MAPK pathway results in increased levels of vascular endothelial growth factor (VEGF), which has an immunosuppressive effect—it inhibits T-cell function and recruitment, increases the recruitment of regulatory T (TREG) cells and myeloid-derived suppressor cells (MDSCs), and hinders the differentiation and activation of dendritic cells.^53,54^ The downstream Akt–mammalian target of rapamycin (mTOR) pathway has also been shown to drive the expression of PD-L1 expression in non-small cell lung cancer.^55^

The PI3K pathway (including AKT–mTOR) is altered in ~59% of oesophageal SCC and 76% of adenocarcinomas.^12^ As noted above, this pathway has been shown to regulate tumour PD-L1 expression^55^ and pathway inhibition has been shown to enhance the infiltration of CD8^+^ T cells.^56^ Loss of expression of phosphatase and tensin homologue (PTEN), a negative regulator of PI3K signalling, results in the activation of PI3K, which correlates with ICI resistance in melanoma.^56^ Although somatic PTEN mutations are uncommon in oesophageal cancer, alterations in PTEN expression commonly occur in oesophageal adenocarcinoma. In a study of 117 resected oesophageal adenocarcinomas, 38% showed absent or markedly reduced PTEN staining by immunohistochemistry.^57^

Activation of WNT–β-catenin signalling in tumour cells is frequently associated with poor spontaneous T-cell infiltration across most human cancers,^58^ and this pathway is aberrantly activated in 30–50% of gastric cancer tissues.^59^ Work by the OCCAMS group suggests a three-way association between the activation of Wnt, hypermutation and the loss of immune signalling genes.^19^ Activation of the WNT–β-catenin pathway is essential for T-cell differentiation, effector function and migration.^60^ Through its main transcription factor, T-cell factor (TCF-1), this pathway promotes the differentiation of naïve CD8 cells into memory cells rather than effector cells, reducing the immediate anti-tumour effect.^58^ It also plays a role in restricting T cells from the immediate TME, thereby contributing to the ability of the tumour to evade the immune response. Analysis of TCGA revealed that activating mutations in β-catenin signalling molecules were enriched three-fold in non-T-cell-inflamed tumours relative to T-cell-inflamed tumours.^61^ With a T-cell-inflamed TME associated with a better response to ICIs, this evidence supports a role for β-catenin in ICI resistance.

IFN-γ plays a key role in the function of effector T cells, with reports of both positive and negative effects on anti-tumour immune responses.^62^ Tumour-specific T cells produce IFN-γ on recognising their respective antigen on tumour cell surfaces or via an antigen-presenting cell, which results in enhanced tumour antigen presentation, recruitment of immune cells and direct anti-proliferative and pro-apoptotic effects on cancer cells.^63^ IFN-γ can also induce the expression of PD-L1 in gastric cancer.^64^ In gastric cancer cell lines, IFN-γ given with PD-L1 antibodies had an enhanced effect compared with PD-L1 antibody monotherapy,^64^ while in clinical samples PD-L1 expression is significantly associated with intra-tumoural IFN-γ and stromal CD8^+^ T cells.^65^ Taken together, these results suggest that gastric cancer patients with constitutively high levels of IFN-γ should be more susceptible to ICIs than patients with low levels. Consequently, tumour cells might downregulate or mutate molecules involved in the IFN-γ signalling pathway.^66^

#### The TME

The function of T cells and their presence in the TME are key elements of the immune response to cancer. A T-cell-inflamed TME improves the efficacy of immune checkpoint blockade, whereas non-T-cell-inflamed tumours rarely benefit.^67^ Within the TME, cells surrounding the tumour can influence ICI resistance mechanisms. These include TREG cells, MDSCs, tumour-associated macrophages (TAMs) and cancer-associated fibroblasts (CAFs),^68^ which all exist within the dynamic operations of the immune system. Their responses are tightly regulated via chemokine-mediated negative-feedback systems.

The presence of TREG cells is common in many human tumours, including gastro-oesophageal cancers, and higher levels of this cell type are associated with a poorer prognosis.^69^ TREG cells have a vital role in maintaining self-tolerance,^70^ using inhibitory cytokines (e.g. IL-10, transforming growth factor β (TGF-β) and IL-35^71^) to suppress T-cell responses, and also play a role in neoangiogenesis, which facilitates metastasis.^72^ Depletion of TREG cells in murine models has been shown to restore and enhance anti-tumour immunity,^73,74^ although this approach increases the potential for the development of autoimmune disorders.^75^ The ability to increase the ratio of effector T cells compared with TREG cells is associated with a positive response to ICIs,^76^ thereby supporting a role for TREG cells in mediating resistance to ICI.

MDSCs are immature myeloid cells that play a key role in the suppression of both the innate and adaptive immune response and in promoting angiogenesis, tumour cell invasion and metastasis.^77^ Their presence is associated with more advanced stage and poorer prognosis, in gastric and oesophageal cancers specifically.^78^ MDSCs are regulated by a complex network of signalling molecules, including IL-6, IL-10, granulocyte colony-stimulating factor (G-CSF), IFN-γ and VEGF, which control MDSC recruitment and activation in the TME.^79^ These molecules appear to signal primarily through the PI3K–Akt and Janus kinase (JAK)–signal transducer and activator of transcription (Stat) pathways.^80^ The C–C motif chemokine ligand 2 (CCL2)– C–C motif chemokine receptor 2 (CCR2) pathway also plays a role in MDSC recruitment in gastric cancer.^81^ The presence of MDSCs appears to predict reduced efficacy of ICI therapy possibly as a consequence of their role in TREG cell expansion, inhibition of effector T cells via CD40, TFG-β and IL-10 and inhibition of natural killer (NK) cell function.^82^ Therefore, reducing the numbers of MDSCs, preventing their recruitment or reprogramming their function could enhance the response to ICIs.

Macrophages that populate the TME (TAMs) can be classified as ‘classically activated’ (M1 or type 1) and ‘alternatively activated’ (M2 or type 2) on the basis of their surface molecules, cytokine profile and metabolism.^83^ M1 macrophages promote a pro-inflammatory, anti-tumour immune response,^84^ whereas M2 macrophages promote tumorigenesis. M2 macrophages also play a key role in metastasis by producing growth factors and proteolytic enzymes and triggering various inhibitory immune checkpoints in T cells.^84^ Higher frequencies of M2 TAMs are associated with poor prognosis in a range of cancers, including gastric and oesophageal cancers.^85–87^ In gastro-oesophageal cancer, increased M2 TAM infiltration is associated with increased PD-L1 expression and therefore may be associated with increased efficacy of ICIs.

CAFs comprise one of the most abundant components of the TME, contributing to the extracellular matrix structure of tumour cells as well as to tumorigenesis. Their presence correlates with poorer outcomes in both oesophageal adenocarcinoma and gastric cancer.^88,89^ CAFs secrete various chemokines that downregulate both the innate and adaptive immune responses;^90^ for example, secretion of TGF-β by CAFs increases the recruitment of MDSCs and TREG cells while inhibiting the function of NK cells, dendritic cells and CD8^+^ T cells.^83^ As ICI therapy requires an immune response, the presence of CAFs has been associated with a poorer response to ICIs.^91^

### Acquired resistance

Although dramatic and sustained responses to ICIs have been seen across a range of tumour types, not all such responses are maintained. Patients who initially respond to ICIs but after a period of time progress are said to have acquired resistance. Acquired resistance can occur owing to a loss of T-cell function, such as altered antigen presentation and IFN-γ signalling, development of tumour or β2-microglobulin (β2M) mutations, and an evolving immune response.

#### Loss of T-cell function

Changes in the functionality of anti-tumoural T cells have been observed in patients with advanced melanoma who underwent adoptive T-cell transfer, with patients who relapsed showing a lack of the cytotoxic activity that was initially observed.^92^ This loss of T-cell response can result from a reduction in antigen presentation, particularly owing to the loss or mutation of β2M.^93^ β2M is a key component of MHC class I molecules and its genetic deficiency in embryonic stem cells results in their failure to be recognised by CD8^+^ T cells.^94^ Similarly, tumour β2M deficiency predicts poor outcomes in several cancers and also predicts resistance to ICI in non-small-cell lung cancer and melanoma.^95^ Interestingly, β2M mutations have been demonstrated following progression on anti-PD1 therapy in gastro-oesophageal adenocarcinoma, but a β2M mutation at baseline does not exclude an initial ICI response.^96^ This supports the concept that acquired resistance is likely a multifactorial process.

Mutation of JAK1/2 is also a potential mechanism of acquired resistance to ICIs.^95^ Loss of function of JAK1/2 results in the absence of IFN-γ-mediated signalling and thus tumour immune evasion. This may be relevant to gastro-oesophageal cancer as—IFN-γ signalling through the cGAS/STING pathway is associated with increased PD-L1 expression and better outcomes with chemotherapy in the neoadjuvant setting.^65^

#### An evolving immune response

The immune response is constantly evolving and a balance exists between pro-inflammatory and inhibitory pathways. The goal of ICI therapy is to stimulate the pro-inflammatory response. Multiple T-cell inhibitory checkpoints exist within the TME of oesophageal adenocarcinoma including those induced by PD-L1, PD-L2, LAG-3, IDO-1, CLTA4 and TIM-3.^97^ IDO-1, for example, is present in ~20% of oesophageal cancer specimens and has been shown to correlate with immune tolerance and poorer outcomes,^98^ whereas in melanoma, it appears that CD8^+^ T cells can drive upregulation of inhibitory checkpoint molecules, probably as part of an autoregulatory negative-feedback mechanism.^99^ We therefore conclude that acquired resistance to ICI therapy in gastro-oesophageal cancer could emerge if the co-existing inhibitory pathways are upregulated, thus negating the pro-inflammatory effect of ICIs.

### Monitoring mechanisms of resistance

The relationship between the tumour and the immune system is constantly evolving and it is therefore vital that changes in tumour genetics are monitored over time. Comprehensive longitudinal assessment of patient and tumour biospecimens during treatment is an emerging strategy. This strategy, which can make use of serial circulating tumour DNA (ctDNA) samples or serial biopsy specimens, was adopted by the Personalised Antibodies for Gastro-Esophageal Adenocarcinoma (PANGEA) study.^100^ Such an approach used longitudinal biopsy samples in melanoma to demonstrate that adaptive immune signatures in samples obtained early during the course of treatment were highly predictive of the response to ICIs. Importantly, analysis of the specimens enabled the identification of potential mechanisms of therapeutic resistance.^101^

A longitudinal approach is of great importance for gastro-oesophageal cancer given the heterogenous nature of the disease and the development of co-existing clones with differing mutational patterns both before and during treatment, as demonstrated by a 2020 publication that reported on the use of whole-genome sequencing (WGS) and the phylogenetic analysis of 388 samples across 18 individuals with metastatic oesophageal adenocarcinoma.^102^ The tumour samples were obtained either during surgery or from warm autopsy. Analysis revealed multiple subclones which each appeared to seed multiple metastatic sites; these subclones seemed to have originated from the primary site. This development of subclones has been termed ‘clonal diaspora’. The spatial discordance between the primary lesion and metastatic sites was also observed in tumour samples from the PANGEA trial.^103^

It is also important to note that gastro-oesophageal adenocarcinoma appears to display both temporal and spatial heterogeneity in terms of TMB and PD-L1 expression before and after chemotherapy.^104,105^ Maron et al.^105^ found that the genomic landscape identified by ctDNA next-generation sequencing (NGS) was similar, but not identical, to tumour NGS suggesting that using ctDNA alongside tumour NGS may provide a mechanism to identify early and target intra-patient heterogeneity. The development of subclones and the reported changes in mutational signature, TMB and PD-L1 expression induced by treatment are clinically relevant as they might indicate a change in the signalling pathways and driver mutations, thus potentially altering the immune environment and the response to cancer therapy.

Obtaining serial biopsy samples can be challenging for numerous reasons, including patient preferences and the lack of an accessible metastatic site to target. One solution might lie in the use of ctDNA, which could also facilitate monitoring of the response to treatment. Studies of gastro-oesophageal cancer and in other tumour groups have shown that ctDNA could help to identify patients with poorer prognosis disease at baseline, monitor response to therapy and elucidate emerging resistance mechanisms.^105,106^

## Current strategies to overcome resistance to ICIs in patients with advanced gastro-oesophageal cancer

**Fig. 3:**
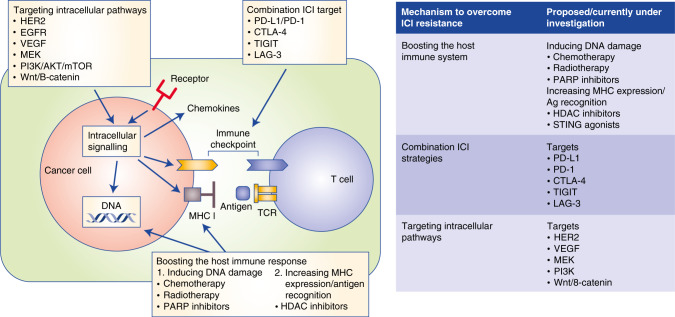
Current/proposed strategies to overcome resistance to immune checkpoint inhibitors. The figure depicts the proposed strategies—targeting intracellular pathways, combination ICI and boosting the host immune system. Potential specific targets are also highlighted. EGFR epidermal growth factor receptor, VEGF vascular endothelial growth factor, MEK mitogen-activated protein kinase kinase, PI3K phosphoinositide 3-kinases, AKT protein kinase B, mTOR mammalian target of rapamycin, β-cat β-catenin, PD-L1 programmed death ligand 1, PD-1 programmed cell death protein 1, CTLA-4 cytotoxic T lymphocyte antigen 4, TIGIT T-cell immunoreceptor with Ig and ITIM domains, LAG-3 lymphocyte-activation gene 3, MHC major histocompatibility complex, TCR T-cell receptor.

An increasing knowledge of the mechanisms that underlie ICI resistance is driving efforts to establish new therapeutic strategies to improve response rates and outcomes in patients with gastro-oesophageal cancer. If, as proposed by Powers and colleagues,^107^ cancer has an ‘immune set-point’—an equilibrium between factors that promote or suppress anti-cancer immunity—then the goal of overcoming ICI resistance is to turn an immunologically ‘cold’ TME into a more inflamed ‘hot’ state by increasing the number of effector T cells and the presentation of neoantigens while dampening immune suppression by regulatory T cells and co-existing immune checkpoints. Most patients with gastro-oesophageal cancer are CIN, with a low immune signature expression, which explains why response rates to single-agent therapy are low.^22^ Consequently, the focus has turned to combination therapies using both novel and established agents (Fig. 3).

### Boosting the immune response

Chemotherapy can be used to increase the TMB via DNA damage, with a subsequent increase in antigen presentation and, therefore, in immune response. Chemotherapy (including platinum agents) also has a role in reprogramming the immunosuppressive effect of MDSCs by altering their sensitivity to apoptosis.^108^ An example of where this has been trialled is the KEYNOTE-062 study, in which pembrolizumab was used in conjunction with chemotherapy for the treatment of patients with advanced gastric or gastro-oesophageal junction adenocarcinoma.^27^ The same principle of inducing DNA damage applies to the use of inhibitors of poly(ADP ribose) polymerase (PARP) and radiotherapy—chemoradiation increases the TMB and exposes antigens^109^—and the use of ICIs with chemoradiation in resectable gastric cancer is currently under investigation (NCT03776487).

Novel approaches to boosting an immune response include the use of histone deacetylase inhibitors (HDACi). HDACi increase the expression of both MHC class I and II and thus presentation of tumour-associated antigens.^110^ This results in enhanced activation of a cytotoxicity T-cell response.

### Combining immune therapy regimens

With the knowledge that negative immune checkpoints are upregulated as part of acquired resistance, combination immune therapy regimens are being tested in gastro-oesophageal cancer. For example, CheckMate 032 investigated the combination of nivolumab plus ipilimumab in patients with metastatic gastro-oesophageal cancer.^37^ Although the Phase 2 non-randomised study showed promise in the first-line setting, the drug combination in the subsequent Phase 3 CheckMate-649 study was terminated early. The reason for this is not yet public, but is likely either due to lack of efficacy, increased toxicity or both. The challenge of combination strategies is to balance efficacy with the increase in immune-related adverse events, particularly in a population that is often older with multiple co-morbidities.

### Targeting intracellular signalling pathways

Cellular signalling by tumour cells can also be targeted, with HER2, VEGF and MEK providing promising targets.

Overexpression or amplification of HER2 is common in oesophageal adenocarcinoma (30% amplification in TCGA; 32.2% HER2-positive gastro-oesophageal junction in TOGA screening cohort).^14^ The HER2/ERBB2 receptor monoclonal antibody trastuzumab not only prevents receptor dimerisation and, thus, downstream signalling, but also interacts with the innate immune system to recruit effector T cells.^111^ HER2-specific antibodies can also trigger NK-cell-mediated antibody-dependent cellular cytotoxicity.^112^ These results provide a biological rationale to combine anti-HER2 therapy with ICI in gastro-oesophageal cancer and are supported by data from two Phase 2 trials using trastuzumab and margetuximab, respectively, with pembrolizumab.^113,114^ These combinations are the focus of the ongoing KEYNOTE-811 (NCT03615326) and MAHOGANY (NCT04082364) studies.

VEGF signalling is involved in dampening the immune response by inhibiting T-cell recruitment, effector T-cell function and dendritic cell maturation as well as by stimulating TREG cells and MDSCs. Inhibiting the VEGF receptor in combination with the use of ICIs could therefore increase response rate. This strategy was effective in the IMbrave150 study in hepatocellular cancer.^115^ As VEGF signalling often promotes MAPK pathway activation, inhibitors of MEK and PI3K might also be of benefit.^116^ Similarly, the key role and common mutations of the Wnt–β-catenin pathway in gastro-oesophageal cancer indicate this pathway could also be targeted.

Some of these pathways are currently being investigated in a Phase 1b/2 umbrella trial of chemotherapy and the PD-L1 inhibitor atezolizumab with ramucirumab (VEGF inhibitor), cobimetinib (MEK inhibitor), tiragolumab (anti-TIGIT; TIGIT is another immune checkpoint protein), linagliptin (an inhibitor of dipeptidyl peptidase 4 (DPP-4)), BL-8040 (a CXCR4 antagonist) or PEGPH20 (PEGylated recombinant human hyaluronidase) (NCT03281369). DPP-4 plays a role in lymphocyte migration,^117^ CXC chemokine receptor 4 (CXCR4) promotes metastatic homing in HER2-positive oesophageal cancer^118^ and PEGPH20 is a stromal modifying agent that depletes hyaluronan and thus alters biophysical and molecular signalling pathways.^119^

## Conclusions

ICIs have been transformative, with sustained responses seen across a range of tumour types. However, the majority of patients with gastro-oesophageal cancer fail to respond to ICI therapy, and many of those who initially respond progress eventually. To broaden applicability, we need to understand and address primary and acquired resistance. Resistance to ICIs is complex and multifactorial, involving tumour and patient factors in a bidirectional interaction. So far, the mechanistic understanding of resistance is incomplete. However, resistance mechanisms seen in other tumour types have provided many hypotheses that require further investigation in the context of gastro-oesophageal cancer.

Combination therapies have been the main approach for current Phase 3 clinical trials, but there has been limited success so far on combining chemotherapy with an ICI or with sequential therapy. In order to tackle the problem of ICI resistance, we need a biology-first approach, followed by precision immunotherapy. This is a key challenge for early-phase trials.

## Data Availability

Not applicable.
